# Intestinal microbiota regulates naïve lymphocyte migration in Peyer’s patches

**DOI:** 10.3389/fimmu.2025.1717788

**Published:** 2025-12-01

**Authors:** Johanna Kabbert, Anne Kaminski, Oliver Pabst, Milas Ugur

**Affiliations:** Institute of Molecular Medicine, Rheinisch-Westfälische Technische Hochschule Aachen, Aachen, Germany

**Keywords:** microbiota, antibiotics, Peyer’s Patches, lymphocyte, migration, intestine, FTY720, 16S rRNA sequencing

## Abstract

Peyer’s patches (PPs) are lymphoid organs in the small intestine that serve as inductive sites for both humoral and cellular immune responses against the microbiota, food antigens and pathogens. PPs in germ-free mice are small and hypocellular, highlighting the importance of the microbiota in regulating PP size. However, it is unclear whether this regulation reflects changes in immune cell activation and germinal center responses or alterations in lymphocyte migration kinetics. In this study, we modified the composition and density of the microbiota using antibiotics to investigate the corresponding changes in PP cellularity. We demonstrated that distinct microbiota compositions can result in a reduction in PP size. This reduction was limited to PPs in the distal small intestine, and it reached germ-free levels after only three days of antibiotic treatment. Reduction in PP size was largely due to changes in lymphocyte circulation kinetics in PPs. Lymphocyte egress blockade using the functional S1PR1 antagonist FTY720 prevented the antibiotic-induced decrease in PP cellularity and the entry of naïve lymphocytes into PPs was reduced in antibiotic-treated mice. Our findings uncover a previously unrecognized role for the microbiota in regulating the migration of naïve lymphocytes into PPs, which has implications for the modulation of adaptive immune responses in the intestine.

## 1 Introduction

The intestinal microbiota has far-reaching local and systemic effects on physiology, metabolism and immunity. While some of these effects can be attributed to a specific bacterial species (e.g. induction of IL-17 producing T cells by segmented filamentous bacteria (SFB)), others arise from the collective activities of several bacterial species (e.g. induction of regulatory T cells by microbial metabolites such as short-chain fatty acids) (1, 2). Conversely, both the innate and adaptive immune systems also modulate the composition of the microbiota via antimicrobial peptides and the induction of secretory immunoglobulins against specific members of the microbiota (3, 4). These interactions result in a complex and dynamic interplay between the microbiota and the host immune system, particularly in the intestine.

Peyer’s patches (PPs) are key sites in the regulation of intestinal immunity where adaptive immune responses to the microbiota, pathogens and food antigens are induced (5–7). Like lymph nodes (LNs), PPs are secondary lymphoid organs (SLOs) which develop during gestation via a defined developmental program and naïve lymphocytes circulate through LNs and PPs while searching for their cognate antigens. However, several features distinguish PPs from LNs. PPs lack afferent lymphatics, and intestinal content is directly sampled from the gut lumen via a subepithelial dome that is enriched in antigen-presenting myeloid cells (5–7). Moreover, unlike LNs, PPs constitutively have germinal centers (GCs) under homeostatic conditions in both colonized and germ-free (GF) mice, which fuels the intestinal plasma cell pool by producing high numbers of IgA^+^ plasmablasts (8).

Germ-free and antibiotic-treated mice have smaller PPs compared to conventionally reared mice. This finding has been ascribed to underdeveloped or shrunken GCs in the PPs of these mice (9–11). However, lymphocytes in GCs represent only a fraction of all lymphocytes in a PP, and we previously reported that PP size is also regulated by changes in immune cell migration during Salmonella infection (12). We thus speculated that changes in GCs alone might not account for the dramatic decrease in PP size in GF and antibiotic-treated mice.

In this study, we used several different antibiotic combinations to manipulate the microbiota and analyzed their effects on PP cellularity. We show that the antibiotic-induced decrease in PP cellularity is a rapid process as the cell numbers in PPs reached GF levels after only three days of antibiotic treatment. Moreover, antibiotics reduced PP cellularity only in distal PPs, emphasizing the local effects of the microbiota in regulating SLO cellularity. 16S rRNA sequencing of intestinal contents revealed that at least two distinct microbiota compositions can reduce PP size and neither total bacterial density nor the presence of SFB were predictive of PP cellularity. Surprisingly, reduction in PP size involved a strong reduction in naïve lymphocyte numbers for both T cells and B cells. This decrease was due to impaired migration of lymphocytes in PPs and blocking lymphocyte egress during antibiotic treatment restored PPs, cellularity. Our results reveal a previously unrecognized role of the microbiota in regulating naïve lymphocyte circulation in PPs, which might pave the way for new approaches in modulating mucosal immune responses.

## 2 Results

### 2.1 Intestinal microbiota modulates PP size primarily in the distal small intestine

**Figure 1:**
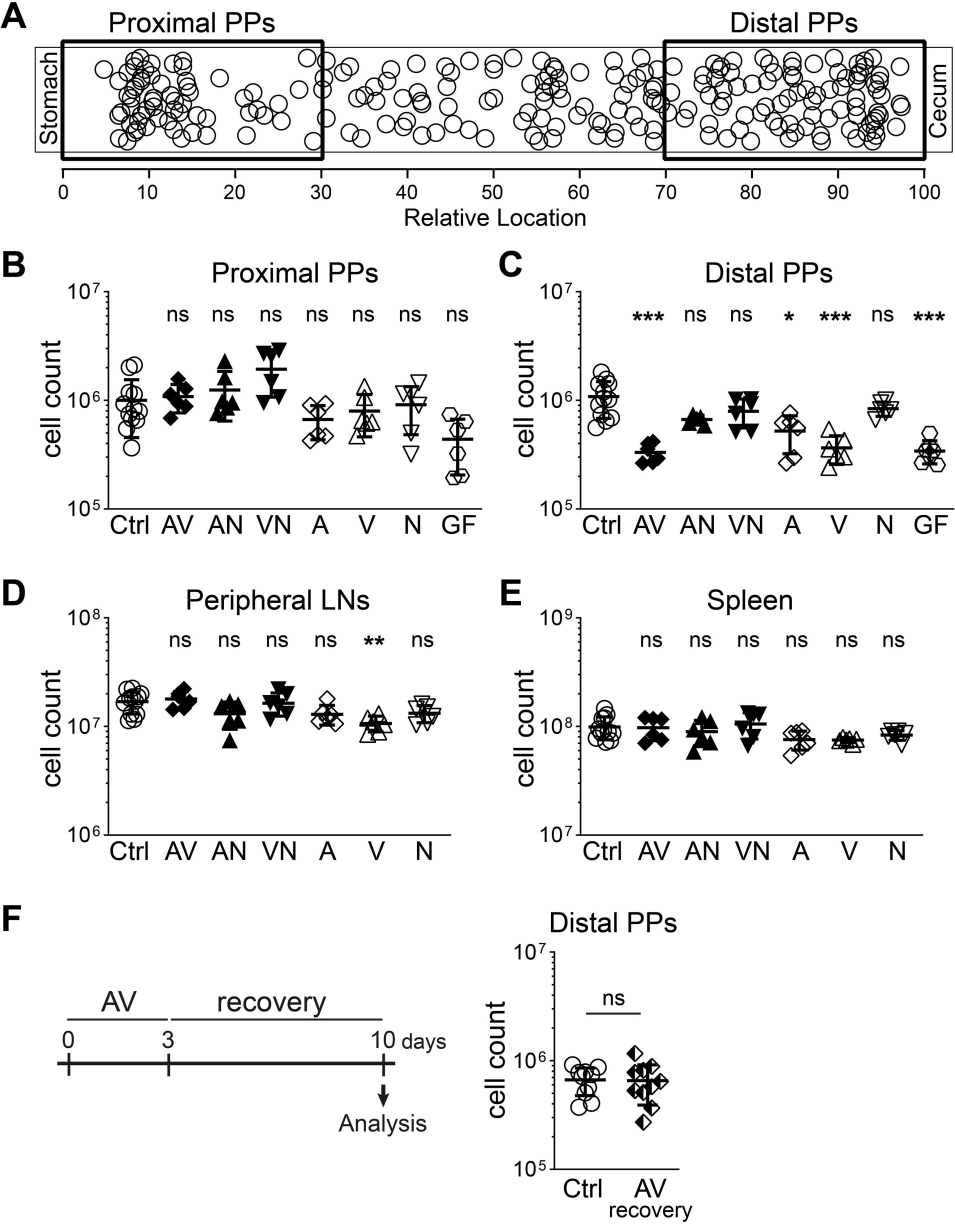
Short-term antibiotic treatment reduces cell numbers only in distal PPs. **(A)** Diagram depicting relative locations of individual PPs along the small intestine of WT mice (n = 30 mice, each circle represents a single PP). **(B–E)** Cell counts in the lymphoid organs of WT mice that are treated with different combinations of antibiotics [Ampicillin (A), Vancomycin (V), Neomycin (N)] for three days, untreated (Ctrl) or germ-free (GF). **(B, C)** Average cell counts for a single PP among proximal **(B)** or distal **(C)** PPs in the small intestine. **(D, E)** Cell counts in six skin-draining peripheral LNs **(D)** or the spleen **(E)**. **(F)** WT mice were treated with AV antibiotics for 3 days, afterwards co-housed with control mice for 7 days without antibiotics and analyzed on day 10. **(B–E)** n = 5–12 mice per group in 4–5 independent experiments, mean ± SD, **(F)** n = 10 mice per group in 2 independent experiments, mean ± SD; one-way ANOVA Kruskal-Wallis test with Dunn’s multiple comparisons test, statistical significance reported as compared to untreated controls **(B-E)**, Mann-Whitney test **(F)**, *P<0.05; **P<0.01; ***P<0.001; ns, not significant.

In mice, 6–12 macroscopically visible PPs are distributed along the anti-mesenteric side of the small intestine (SI). We found that PPs were not homogeneously distributed along the SI; rather, they were enriched in the proximal and distal parts while PP density was lower in the middle SI segments (**Figure 1A**). The microenvironment along the SI changes drastically from proximal to distal regions due to factors such as microbial composition, digestive enzymes, nutrient reabsorption, pH, and oxygenation levels (13, 14). Moreover, PPs vary in size and in the number of follicles they contain, complicating the quantification of PP cellularity and the evaluation of the effects of microbiota on PP size. To account for this intrinsic heterogeneity in the PP anatomy, we pooled PPs from the first 30% of the small intestinal length (starting from the stomach) as proximal PPs and the last 30% (ending at the cecum) as distal PPs (**Figure 1A**). We quantified PP size (i.e. cellularity) by dividing the total cell count of each pool by the number of PPs in that pool to calculate the average cell count per PP.

To investigate the effects of intestinal microbiota on PP size, we treated wild type (WT) mice with the antibiotics ampicillin (A), vancomycin (V), neomycin (N), either alone or in combinations, for three days in drinking water ad libitum. These antibiotics are commonly used to manipulate the composition of the intestinal microbiota and do not affect water consumption in mice (15). Three days after antibiotic treatment, we did not observe any significant changes in the cellularity of proximal PPs (i.e. average cell count per PP) (**Figure 1B**). In contrast, there was a significant reduction in distal PP size in AV, A-only and V-only treated mice compared to control mice, indicating local regulation of PP cellularity upon changes in the microbiota (**Figure 1C**). Surprisingly, distal PPs of AV or V-only treated mice were of comparable size to the ones of GF mice already after three days of treatment, suggesting a rapid regulation of PP size by the microbiota (**Figures 1B, C**). Of note, we did not detect a significant decrease in peripheral LNs or the spleen, except for a minor reduction for peripheral LNs after V-only treatment (**Figures 1D, E**). These results demonstrate that intestinal microbiota has fast, local and substantial effects on distal PP size while the size of proximal PPs are largely unaffected by the antibiotic treatment. Notably, only specific antibiotic combinations reduced distal PP cellularity, suggesting that distinct microbial communities differentially influence PP size. The antibiotic-induced reduction in distal PP size was a reversible process as the withdrawal of the antibiotic treatment and cohousing with control mice for seven days restored the PP cellularity in previously antibiotic-treated mice (**Figure 1F**). This indicates that PP cellularity is dynamically regulated according to the transient changes in the intestinal microbiota.

### 2.2 Distinct microbiota compositions can induce a reduction in PP size

**Figure 2:**
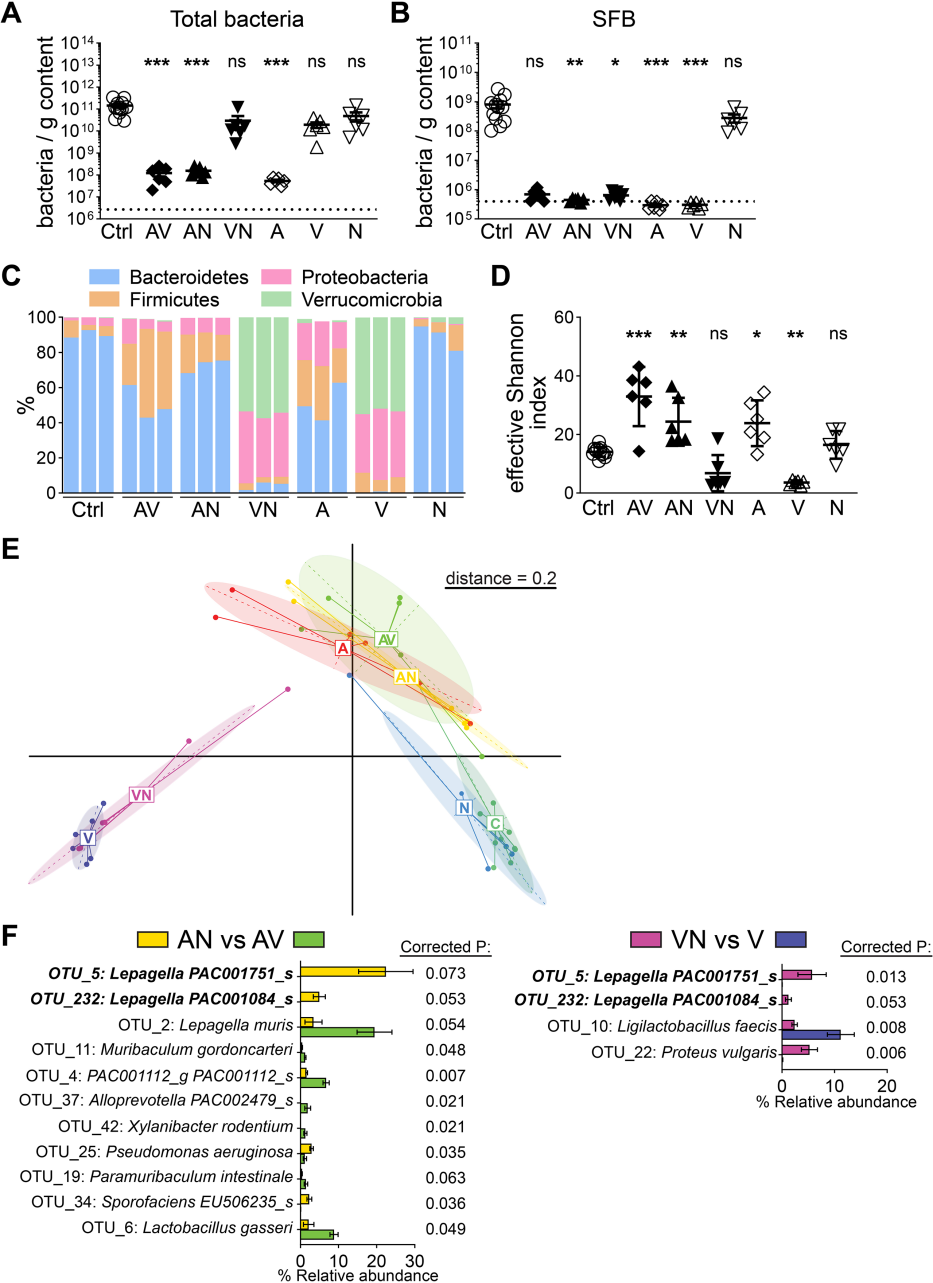
Effects of different antibiotic treatments on the intestinal microbiota. **(A, B)** Density of total bacteria (Eubacteria) **(A)** or segmented filamentous bacteria (SFB) **(B)** in the distal small intestinal content of mice that were treated with different combinations of antibiotics [Ampicillin (A), Vancomycin (V), Neomycin (N)] for three days or left untreated (Ctrl). **(C–F)** Distal small intestinal contents of mice that were treated with different combinations of antibiotics (Ampicillin (A), Vancomycin (V), Neomycin (N)) for three days or left untreated (Ctrl) were analyzed for bacterial 16S rRNA sequences. **(D)** Abundance of major bacterial phyla in 3 representative mice per group. **(C)** Effective Shannon indices for microbial diversity. **(E)** Multidimensional scaling (MDS) analysis of microbial diversity using a generalized UniFrac matrix. **(F)** Comparisons of operational taxonomic unit (OTU) relative abundances between AN vs AV (left) and VN vs V (right). OTUs that have P ≤ 0.05 and corrected P ≤ 0.1 are displayed. **(A–F)** n = 6–12 mice per group in 4 independent experiments, mean ± SEM **(A, B, F)**, mean ± SD **(D)**; one-way ANOVA Kruskal-Wallis test with Dunn’s multiple comparisons test **(A, B)**, one-way ANOVA with Dunnett multiple comparisons test **(D)**, Mann-Whitney test with Benjamini-Hochberg multiple comparisons correction **(F)** statistical significance reported as compared to untreated controls **(A, B, D)**, corrected P values are shown **(F)** *P<0.05; **P<0.01; ***P<0.001; ns, not significant, dotted lines indicate average lower limit of quantification.

Next, we investigated antibiotic-induced changes in the intestinal microbiota to delineate factors underlying the reduction in PP size. Three days after antibiotic treatment, total bacterial densities were reduced in the distal SI contents of AV, AN and A-only treated mice, demonstrating the strong and rapid effects of ampicillin on total bacterial loads (**Figure 2A**). However, there was no significant difference between the control and the VN, V-only and N-only groups (**Figure 2A**). Notably, the effects of antibiotics on bacterial load and PP cellularity differed. AN treated mice had low bacterial density but normal-sized PPs while V-only treated mice had high bacterial density but small PPs (**Figures 1C**, **2A**). This suggests that total bacterial density alone is not the primary determinant of PP size.

Follicle associated epithelial cells of PPs can be closely associated with SFB and the stimulation of B and T cell responses in PPs have been shown to depend on SFB (10, 16, 17). Therefore, we analyzed SFB densities in the distal SI content after three days of antibiotics treatment and observed a substantial decrease after all treatments except for N-only treatment (**Figure 2B**). Importantly, AN and VN treatments did not cause a significant reduction in PP size despite a reduction in SFB densities. This indicates that SFB densities in the SI are not the chief determinant of PP size (**Figures 1C**, **2B**).

Since densities of total bacteria or SFB did not correlate with PP size, we further characterized the changes in the intestinal microbiota compositions after antibiotic treatment by 16S ribosomal RNA (rRNA) sequencing. AV, AN, or A-only treatments led to an increased relative abundance of Firmicutes and Proteobacteria in the distal SI content compared to control mice, accompanied by a corresponding relative decrease in Bacteroidetes (**Figure 2C**). Although VN and V-only treatments did not cause a significant decrease in total bacterial density, there was a substantial shift in the microbiota composition with increases in the relative abundance of Verrucomicrobia and Proteobacteria while N-only treatment did not cause any major changes (**Figure 2C**). Furthermore, effective Shannon indices, which represent the abundance of different bacterial species within a microbial community, were only reduced for VN and V-only treatments, indicating that microbial alpha diversity alone cannot predict PP cellularity as AV, A-only and V-only treatments all resulted in a decrease in PP size (**Figures 1C**, **2D**).

Multidimensional scaling analysis of the microbiota composition between the different treatment groups revealed that VN and V-only treated mice clustered together while control and N-only treated mice formed a separate group (**Figure 2E**). However, AV or A-only treated mice did not cluster together with V-only mice despite all these treatments causing a decrease in PP size, indicating that there are at least two distinct broad microbiota compositions that can result in reduced PP cellularity (**Figure 2E**). To identify potentially shared features between these two broad microbiota compositions, we performed pairwise comparisons within these clusters. We compared AN with AV, where AV caused a reduction in PP size, and VN with V-only, where V-only caused a reduction in PP size. In the AN vs AV comparison, we identified 4 operational taxonomic units (OTUs) that were reduced and 7 OTUs that were increased in relative abundance after AV treatment (**Figure 2F**). In the VN vs V-only comparison, we identified 3 OTUs that were reduced and only one OTU that was increased in relative abundance after V-only treatment (**Figure 2F**). Surprisingly, two OTUs were shared in these comparisons and reduction in both of them was associated with reduced PP size. Interestingly, both of these OTUs belonged to the recently described genus *Lepagella* from the family Muribaculaceae, which is known to produce short-chain fatty acids (SCFAs) (18, 19). Together, these findings indicate that multiple microbial compositions can result in reduced PP size and various features of the microbiota may collectively contribute to the regulation of PP cellularity.

### 2.3 Antibiotic treatment reduces naïve lymphocyte numbers in PPs

**Figure 3:**
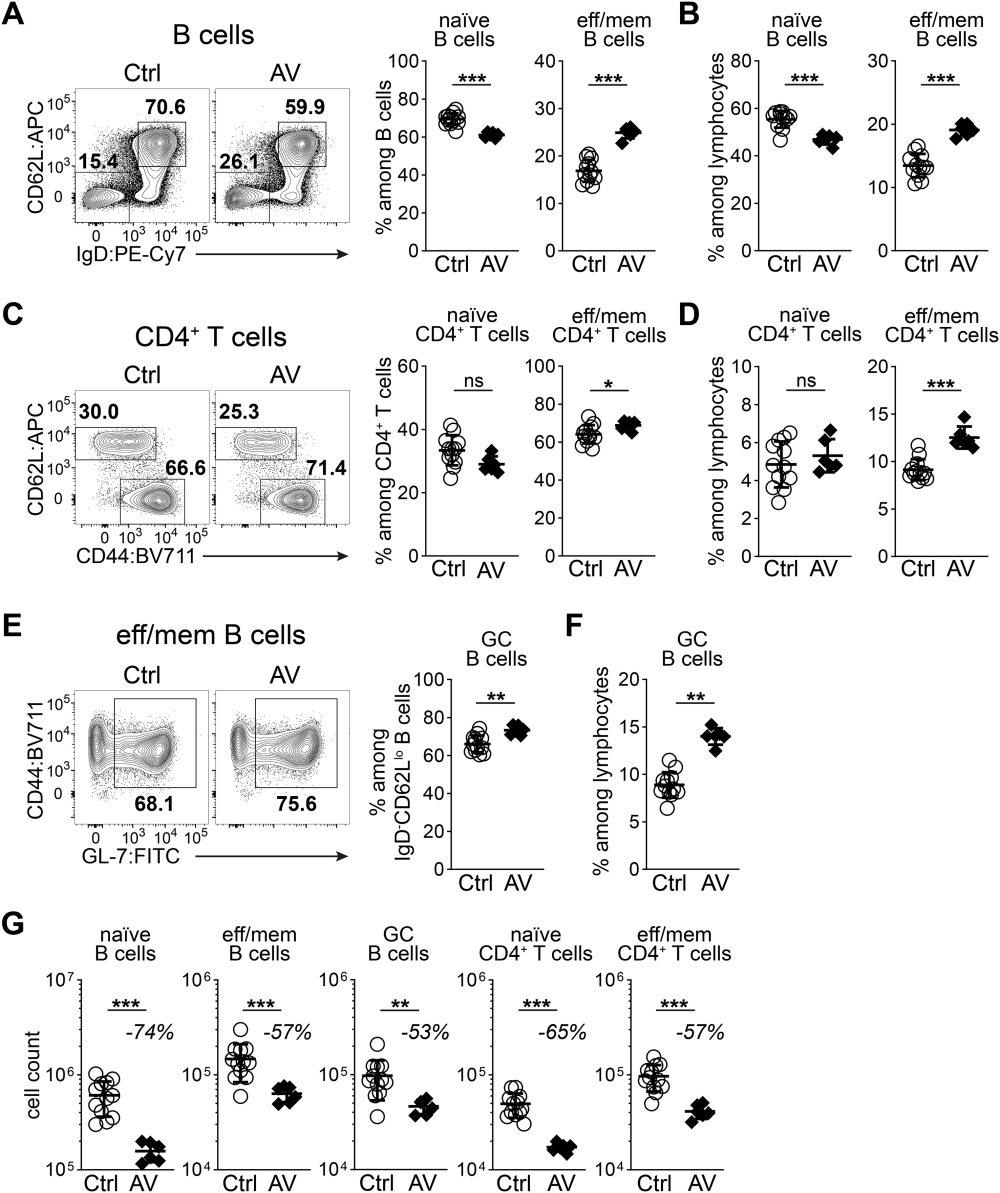
Antibiotic treatment reduces the numbers of both naïve and effector/memory lymphocytes in PPs. **(A-G)** Flow cytometry analysis of distal PPs of untreated (Ctrl) or AV-treated mice after three days. **(A)** Expression of CD62L and IgD among B cells and the frequencies of naïve (IgD^+^CD62L^hi^) and effector/memory (IgD^-^CD62L^lo^) cells among B cells. **(B)** Frequencies of naïve and effector/memory B cells among all lymphocytes. **(C)** Expression of CD62L and CD44 among CD4^+^ T cells and the frequencies of naïve (CD62L^hi^CD44^lo^) and effector/memory (CD62L^lo^CD44^hi^) cells among CD4^+^ T cells. **(D)** Frequencies of naïve and effector/memory CD4^+^ T cells among all lymphocytes. **(E)** Expression of CD44 and GL7 among effector/memory (IgD^-^CD62L^lo^) B cells and the frequencies of GL-7^+^ cells among effector/memory B cells. **(F)** Frequencies of IgD^-^CD62L^lo^GL-7^+^ germinal center (GC) B cells among lymphocytes. **(G)** Average cell counts of naïve B cells, effector/memory B cells, GC B cells, naïve CD4^+^ T cells and effector/memory CD4^+^ T cells in a single PP among distal PPs. Percentage values indicate the average decrease. **(A-G)** n = 6–12 mice per group in 4 independent experiments, mean ± SD; unpaired Student’s t-test **(A-F)**, Mann-Whitney test **(G)**, *P<0.05; **P<0.01; ***P<0.001; ns, not significant.

To study the cellular dynamics underlying the antibiotic-induced reduction in PP size, we focused on AV treatment as a model system. Flow cytometry analysis revealed a reduction in the frequency of naïve cells (IgD^+^CD62L^hi^) and a corresponding increase in effector/memory (i.e. non-naïve) cells (IgD^-^CD62L^lo^) among B cells in distal PPs of antibiotic-treated mice (**Figure 3A**). These shifts in frequencies were also reflected when analyzing frequencies among all lymphocytes in the PPs (**Figure 3B**). We also observed a similar trend of reduction in naïve (CD62L^hi^CD44^lo^) and increase in effector/memory (CD62L^lo^CD44^hi^) CD4^+^ T cell frequencies after AV treatment (**Figures 3C, D**). Furthermore, the frequency of GC (GL-7^+^) B cells among effector/memory B cells and all lymphocytes also increased after AV treatment (**Figures 3E, F**). These changes in frequencies suggest that different lymphocyte subsets are differentially affected by changes in the intestinal microbiota. Notably, the number of cells for all lymphocyte subsets decreased but this decrease was more dramatic among naïve lymphocytes (**Figure 3G**). This in turn caused a relative increase in the frequency of effector/memory B cells and effector/memory CD4^+^ T cells among all lymphocytes although their numbers were also reduced after antibiotic treatment (**Figures 3B, D, G**). These results demonstrate that naïve lymphocyte populations in distal PPs are particularly sensitive to changes in the microbiota. Moreover, the reduction in naïve B cell numbers is a major contributor to the overall reduction in PP size after antibiotic treatment as they constitute >50% of all lymphocytes in PPs.

### 2.4 Antibiotic treatment impairs naïve lymphocyte homing to PPs

**Figure 4:**
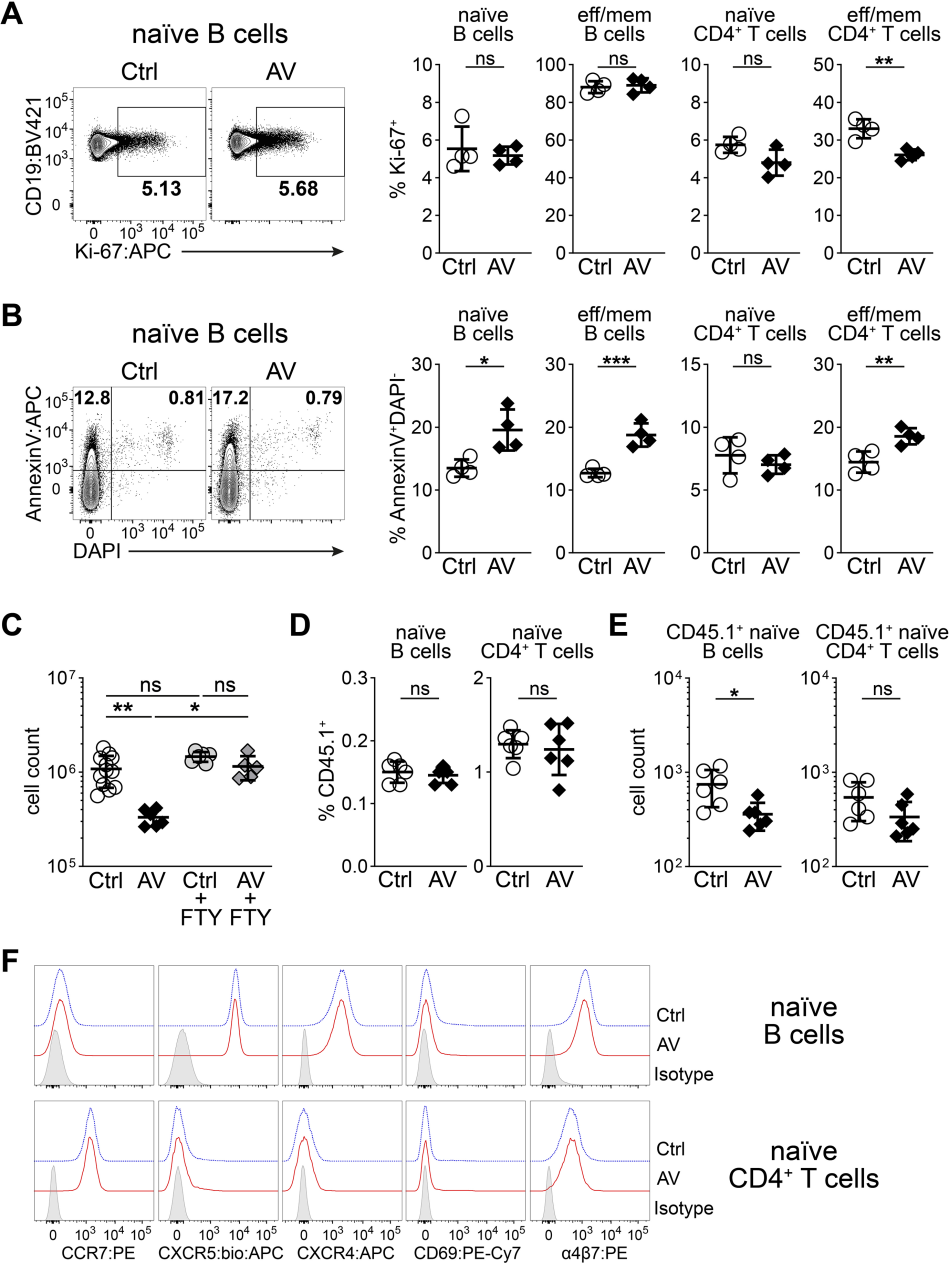
Decreased lymphocyte migration drives the antibiotic-induced reduction in PP cellularity. **(A, B)** Flow cytometry analysis of distal PPs of untreated (Ctrl) or AV-treated mice after 3 days. **(A)** Expression of Ki-67 among naïve B cells and the frequencies of Ki-67^+^ cells among naïve B cells, effector/memory B cells, naïve CD4^+^ T cells and effector/memory CD4^+^ T cells. **(B)** Expression of Annexin V among naïve B cells and the frequencies of AnnexinV^+^DAPI^-^ early apoptotic cells among naïve B cells, effector/memory B cells, naïve CD4^+^ T cells and effector/memory CD4^+^ T cells. **(C)** Average cell counts for a single PP among distal PPs of WT mice treated with FTY720 only (Ctrl+FTY720) or AV+FTY720 in the drinking water for 3 days. The data from **Figure 1** were duplicated for Ctrl and AV groups for visualization and statistical analysis. **(D, E)** CD45.1^+^ lymphocytes were transferred to Ctrl or AV-treated mice and distal PPs were analyzed 2 hours after the transfer. **(D)** Frequencies of CD45.1^+^ cells among naïve B cells and naïve CD4^+^ T cells. **(E)** Average cell counts of CD45.1^+^ naïve B cells and CD45.1^+^ naïve CD4^+^ T cells in a single PP among distal PPs. **(F)** Representative flow cytometry analysis of CCR7, CXCR5, CXCR4, CD69 and integrin α4β7 in naïve (IgD^+^CD62L^hi^) B cells and naïve (CD62L^hi^CD44^lo^) CD4^+^ T cells from the distal PPs of untreated (Ctrl) and AV-treated mice three days after the treatment. **(A, B)** n = 4 pools of 8 mice per group in 2 independent experiments, unpaired Student’s t-test. **(C)** n = 5–6 mice per group in 2 independent experiments, one-way ANOVA Kruskal-Wallis test with the corrected method of Benjamini and Yekutieli. **(D, E)** n = 6 mice per group in 2 independent experiments, **(F)** representative of at least 2 mice in 2 independent experiments; unpaired Student’s t-test **(D)** and Mann-Whitney test **(E)**. **(A-E)** mean ± SD, *P<0.05; **P<0.01; ***P<0.001; ns, not significant.

In principle, lymphocyte numbers in PPs can change because of cell proliferation, cell death and lymphocyte circulation kinetics. To examine if reduced rates of cell proliferation might contribute to the decrease in cell numbers after antibiotic treatment, we assessed cell proliferation using Ki-67 three days after AV treatment. We detected no difference in B cells and only minor reductions among naïve and effector/memory CD4^+^ T cells after antibiotic treatment, suggesting that reduced proliferation rates do not contribute substantially to the reduction in PP size (**Figure 4A**). As an estimate of cell death, we stained for the apoptosis marker Annexin V. In AV-treated mice, we observed higher frequencies of early apoptotic (AnnexinV^+^DAPI^-^) cells among naïve B cells, effector/memory B cells and effector/memory CD4^+^ T cells (**Figure 4B**). This suggests that increased cell death might contribute to reduced cellularity in PPs after AV treatment. Notably, naïve CD4^+^ T cells did not show any increase in cell death despite a decrease in cell numbers, indicating that additional factors might contribute to the reduction in cell numbers after antibiotic treatment (**Figures 3D**, **4B**).

Lymphocytes constantly traffic through PPs and changes in entry and exit rates can substantially alter cellularity in PPs (12). To investigate the influence of lymphocyte circulation in PP size reduction after antibiotic treatment, we used the S1PR1 functional antagonist FTY720, which effectively halts lymphocyte circulation through LNs and PPs due to conjunctional effects of systemic egress blockade and lymphopenia (20). Co-administration of FTY720 with antibiotics effectively prevented the antibiotic-induced reduction in PP size, suggesting that impaired lymphocyte trafficking through PPs is the main driver of decreased cellularity in PPs (**Figure 4C**).

To directly study the effects of antibiotic treatment on lymphocyte homing into PPs, we transferred CD45.1^+^ lymphocytes into control or AV-treated WT CD45.2^+^ mice 2 hours before analysis. Although the frequencies of transferred lymphocytes were similar between groups, we observed lower numbers of transferred naïve lymphocytes after antibiotic treatment, indicating a reduction in lymphocyte entry to PPs (**Figures 4D, E**). Importantly, naïve lymphocytes from control and AV-treated mice expressed comparable levels of migration-related molecules such as CCR7, CXCR5, CXCR4, CD69 and integrin α4β7 in distal PPs (**Figure 4F**). This implies that an impairment in the lymphocyte recruitment capacity of blood endothelial cells in PPs rather than a change in lymphocyte migratory phenotype mediates reduced lymphocyte entry after antibiotic treatment. In summary, these findings identify reduced lymphocyte migration in PPs as the main driver of the decrease in PP cellularity after antibiotic treatment.

## 3 Discussion

Intestinal microbiota has been shown to be an important regulator of PP cellularity, but the focus of these previous analyses had been the GC within PPs (9–11). Here, we show that antibiotic-induced changes in microbiota also affect naïve lymphocyte numbers in PPs, and the overall size reduction in PPs is primarily due to a decrease in naïve lymphocyte recruitment from blood to PPs. The reduction in PP cellularity was a local effect restricted to distal PPs and was induced only by specific antibiotic treatments. Our analysis of the intestinal microbiota in terms of its composition, density and diversity revealed at least two broad microbiota compositions that can induce a reduction in PP size. Interestingly, we identified two OTUs from the *Lepagella* genus that are shared between these microbial compositions and in both cases reduction in the relative abundance of these OTUs were associated with a reduced PP size. The *Lepagella* genus belongs to the Muribaculaceae family, which is known for its ability to ferment dietary fibers and produce SCFAs that can regulate various aspects of mucosal immunity via modulation of epithelial integrity and T cell differentiation (21, 22). However, the relative abundance of other OTUs that belong to the Muribaculaceae family, such as *Lepagella muris* and *Muribaculum gordoncarteri*, increased with the AV treatment. Therefore, further investigations are required to clarify causal relationships between specific *Lepagella* species, SCFA production and reduction in PP size.

In our 16S rRNA microbiota analysis, we used relatively strict criteria (relative abundance ≥0.5 and minimal prevalence ≥0.3) for the identification of potential species associated with a reduced PP size. Therefore, less prevalent members of the microbiota might not have been detected in our analysis. Furthermore, we analyzed the microbiota from the distal SI content, which might cause sampling bias and missing of bacteria localized in different compartments. One candidate taxonomic group of bacteria that may regulate PP size are the members of the Alcaligenes, Achromobacter, Bordetella and Ochrobactrum genera, which reside in lymphoid organs such as PPs (17, 23). These bacteria are reported to modify intestinal immunity via IL-10 signaling and changes in their abundance might not have been detected in our analysis of the microbiota in the distal SI content. Alternatively, regulation of PP size might be a collective effect of several species with a common function, which can be compensated by remaining species after antibiotic-induced changes in microbiota. For example, osmolarity in the intestinal lumen has been recently shown to regulate lymphocyte entry into PPs by modifying the function of high endothelial venules (HEVs) that recruit lymphocytes from blood (24). Therefore, different microbiota compositions might increase osmolarity of the intestinal lumen in a similar manner, causing a reduction in PP size despite distinct microbial communities emerging after treatment with different antibiotics.

We did not observe a change in the expression of chemokine receptors or integrins on naïve lymphocytes in PPs despite their reduced recruitment, suggesting an impaired function of HEVs in PPs upon drastic or sudden changes in the microbiota. Although the differentiation of blood endothelial cells in HEVs is initiated by lymphoid tissue inducer cells, their function is also actively maintained in SLOs by dendritic cells (DCs) via lymphotoxins and vascular endothelial growth factor (25–27). DCs not only present antigens to naïve lymphocytes but also integrate innate signals from their microenvironments to coordinate and direct adaptive immune responses. Furthermore, DCs are continuously replaced by precursors from the bone marrow to constantly update the information on the immunological status of tissues (28). PPs contain resident DC populations that constantly sample luminal antigens (29), positioning DCs as likely candidates for integrating microbiota-derived signals and regulating HEV function to adjust PP cellularity.

As in other SLOs, naïve lymphocytes circulate rapidly through PPs and most of the naïve lymphocytes in PPs are replaced within 12–24 hours (12, 30, 31). Due to this fast turnover, even small changes in the rates of lymphocyte entry or exit can result in dramatic changes in PP cellularity over time. Indeed, cell numbers in a specific LN are highly stable over prolonged periods and a pressure-gated access model based on the release of pressure around HEVs with the egress of lymphocyte via lymphatic vessels has been proposed to balance lymphocyte entry and exit rates (32). This tight coupling of lymphocyte entry and exit rates might explain why we could not detect a difference in the frequencies of newly recruited lymphocytes after antibiotic treatment despite reduced numbers. Alternatively, a decrease in the frequency might not have been detected on day three after antibiotic treatment because the cell numbers in distal PPs had already reached GF levels and the decrease in cellularity was already at its minimum at the analysis time point.

After antibiotic treatment, we observed more pronounced decrease among naïve B cells compared to naïve CD4^+^ T cells in distal PPs, both in total numbers and in the adoptive transfer experiment, which suggests that naïve B and T cell homing into PPs might be differentially affected by the changes in the microbiota. In addition to the shared requirements for CCR7 and CXCR4 in both B and T cells, B cells also rely on CXCR5 for PP homing (33). Moreover, B cells express higher levels of CXCR4 and the gut-homing associated integrin α4β7 compared to naïve T cells. Therefore, alterations in the expression of the chemokines CXCL12 and CXCL13 or of integrin α4β7 ligands such as MAdCAM-1 in PPs upon antibiotic treatment might underlie the differential effects between naïve B and T cells.

In addition to the differences in their homing capacities, B cells also express lower levels of the egress-promoting receptor S1PR1 compared to T cells (34). Naïve B cells also have slower turnover rates in PPs, which might contribute to the more prominent decrease in naïve B cell numbers (30). Although we observed reductions in the homing rates of naïve lymphocytes after antibiotics, increased egress rates may also contribute to the overall decline in naïve lymphocyte numbers. Changes in the microbiota might modulate local S1P gradients by enhancing S1P production by lymphatic endothelial cells or by reducing the S1P degradation in the PP parenchyma, ultimately resulting in a steeper S1P gradient and faster recovery of surface S1PR1 expression in naïve lymphocytes locally in distal PPs (35).

Swelling or hypertrophy of SLOs is a well-known phenomenon associated with inflamed LNs that includes increased lymphocyte entry, reduced egress and the remodeling of the stromal cells in the draining SLO (12, 36, 37). As cognate lymphocytes for a specific antigen are rare cells, this local increase in cellularity in inflamed SLOs increases the probability of finding cognate lymphocytes to facilitate the initiation of adaptive immune responses. Here, we describe a reverse situation; hypotrophy of PPs, in which the cellularity decreases locally in distal SI upon antibiotic treatment. Therefore, a decrease in naïve lymphocyte numbers in PPs might hamper adaptive immune responses against pathogens during antibiotic treatments. Conversely, therapies aiming at selectively reducing lymphocyte homing to PPs might alleviate undesired immune responses in the intestine without compromising immune responses in other organs.

## 4 Materials and methods

### 4.1 Mice

CD45.1^+^ (B6.SJL-PtprcaPepcb/BoyJ) mice were bred and reared at the Animal Facility of Uniklinik RWTH Aachen under specified pathogen free conditions. Wild type C57BL/6N mice were purchased from Charles River, Germany. Germ-free C57BL/6J mice were bred and reared in Central Animal Facility of Hannover Medical School under germ-free conditions. All mice used in the experiments were 8 weeks old females. All experiments were approved by North Rhein-Westphalia State Agency for Nature, Environment and Consumer Protection (Landesamt fur Natur, Umwelt und Verbraucherschutz Nordrhein-Westfalen, LANUV) and performed in accordance with relevant local guidelines and regulations. All experiments were performed in compliance with ethical regulations of German Law for the Protection of Animal Welfare (Tierschutzgesetz).

### 4.2 Antibiotics and FTY720 treatment

Different combinations of antibiotics as indicated in the main text and figures were administered ad libitum in the drinking water for 3 days. The concentrations of antibiotics were as follows: Ampicillin (Sigma) 1 g/L, Neomycin (Sigma) 1 g/L, Vancomycin (Hexal) 0.5 g/L. FTY720 (Sigma) was administered ad libitum in the drinking water at a concentration of 2.5 µg/mL.

### 4.3 Flow cytometry

Single cell suspensions were prepared by meshing LNs (pool of inguinal, axillary and brachial LNs, 6 LNs in total), PPs and spleens through nylon filters in phosphate buffered saline solution containing 3% fetal calf serum (PBS/FCS). Subsequent steps of surface stainings were performed in PBS/FCS. Blocking was performed with PBS/FCS solution containing 5% rat serum. Appropriate combinations and concentrations of antibodies against TCRβ (H57-597), CD3 (17A2), CD4 (RM4-5), CD8α (53-6.7), CD19 (6D5), CD62L (MEL-14), CD44 (IM7), IgD (11-26c.2a), GL-7 (GL-7), CD45.1 (A20), CD45.2 (104), CCR7 (4B12), CXCR5 (2G8), CXCR4 (L276F12), CD69 (H1.2F3), α4β7 (DATK32), Ki-67 (SolA15), and appropriate isotype control antibodies were used for stainings. Antibodies were bought from BioLegend, eBioscience, BD or R&D. Ki-67 staining was performed using Foxp3/Transcription Factor Staining buffer set from eBioscience and it was used according to manufacturer’s instructions. Annexin V staining was performed using Annexin V Apoptosis Detection Kit (BioLegend) and DAPI (4’,6-diamidino-2-phenylindole) according to manufacturer’s instructions. Cell counting was performed using CountBright Absolute Counting Beads (Life Technologies). Average cell counts for PPs were calculated by dividing the total cell number from a pool of PPs to the number of PPs in that pool. All measurements were done on LSR Fortessa (BD) and analyzed using FlowJo software (Tree Star).

### 4.4 RT-PCR for bacterial DNA

Bacterial DNA isolation from intestinal contents was performed with QIAamp DNA Stool Mini kit (QIAGEN). Real-time PCR was performed using a CFX96 Real Time System (BioRad) and SYBR Green detection (Takara) with primers; Eubacteria UniF334 5’- ACTCCTACGGGAGGCAGCAGT -3’, UniR514 5’- ATTACCGCGGCTGCTGGC -3’ (38), Segmented filamentous bacteria 779F 5’- TGTGGGTTGTGAATAACAAT -3’, 1008R- 5’- GCGGGCTTCCCTCATTACAAGG -3’ (39). PCR products were cloned into pGEM-T vector (Promega) and standard curves with serial dilutions were prepared. Number of DNA molecules was calculated using the formula (DNA amount g/[plasmid length in basepairs x 660]) x 6.022 x 10^23^. Lower limit of quantification (LLOQ) was defined as the lowest amount of template DNA that still falls into the linear range of the standard curve. Average LLOQ was calculated using the average amount of intestinal content used in an analysis and displayed in each graph as a dotted line.

### 4.5 16S rRNA sequencing

Metagenomic bacterial DNA isolation from distal small intestinal contents was performed with QIAamp DNA Stool Mini kit (QIAGEN) according to manufacturer’s instructions. 16S rRNA gene V4 regions were amplified (35 cycles) using primers 5’- AATGATACGGCGACCACCGAGATCTACACTATGGTAATTGTGTGCCAGCMGCCGCGGTAA-3’ and 5’-CAAGCAGAAGACGGCATACGAGAT-[Golay Barcode]-AGTCAGTCAGCCGGACTACHVGGGTWTCTAAT-3’ in which [Golay Barcode] represents the multiplex identifier sequence specific for each sample (40, 41). Amplicons were purified with QIAquick Gel Extraction kit (QIAGEN) after agarose gel electrophoresis and quantified using the Quant-iT PicoGreen dsDNA Kit (Invitrogen). Amplicon libraries were sequenced with single barcodes in paired-end mode (2 × 300 nt) using MiSeq sequencer (Illumina). Raw reads were processed using the IMNGS platform (42). Operational taxonomic units (OTUs) were clustered at a sequence identity of 97%. Taxonomic assignment was based on the Ribosomal Database Project (RDP) classifier v2.11 (43) with a confidence cutoff of 80%. Diversity and sample composition analyses were performed using the Rhea pipeline v2.0 in R (44). OTUs with relative abundance ≥0.5 and minimum prevalence ≥0.3 (30%) in at least one treatment groups were considered for the statistical analysis. OTU lineage and taxonomic identity (closest species/strain with a valid name and corresponding 16S rRNA gene sequence identity) were obtained using EZBioCloud (45). All identified OTUs have been previously reported to be typical constituents of the normal (steady state) SPF mouse gut microbiota and the displayed OTUs had similarities ≥ 98.28%.

### 4.6 Cell transfers

5x10^6^ cells from pools of LNs of CD45.1^+^ mice were transferred intravenously into wild type (CD45.2^+^) recipients in PBS and the recipient mice were analyzed 2 hours after the transfer.

### 4.7 Statistics

Statistical analysis was performed using GraphPad Prism software. For comparisons, unpaired Student’s t-test, Mann-Whitney test, one-way ANOVA with Dunnett multiple comparisons test, one-way ANOVA Kruskal-Wallis test with the corrected method of Benjamini and Yekutieli, Kruskal-Wallis test with Dunn’s multiple comparisons or Mann-Whitney test with Benjamini-Hochberg correction were used as described in the figure legends. Data are presented as mean ± SD (standard deviation) or mean ± SEM (standard error of mean) as described in the figure legends. P<0.05 are considered as significant. In all figures; *: P<0.05, **: P<0.01, ***: P<0.001, ns: not significant.

## Data Availability

The 16S rRNA datasets generated and analyzed for this study can be found in the BioProject repository under the accession number PRJNA1304138.
